# Prognostic Value of Long Noncoding RNA SPRY4-IT1 on Survival Outcomes in Human Carcinomas: A Systematic Review and Meta-Analysis with TCGA Database

**DOI:** 10.1155/2020/5868602

**Published:** 2020-11-01

**Authors:** Hai-mei Wang, Hui-jie Li, Jun-zhen Chen, Ke Pei, Qian Zhang, Zhai-dong Liu, Yuan-fu Qi

**Affiliations:** ^1^First Clinical College of Shandong University of Traditional Chinese Medicine, Jinan, Shandong, China; ^2^Department of Oncology, Affiliated Hospital of Shandong University of Traditional Chinese Medicine, Jinan, Shandong, China; ^3^Department of Neurology, Zibo Hospital of Traditional Chinese Medicine, Zibo, Shandong, China; ^4^College of Traditional Chinese Medicine, Shandong University of Traditional Chinese Medicine, Jinan, Shandong, China; ^5^Department of Thyroid Surgery, Weihai Traditional Chinese Medicine Hospital, Weihai, Shandong, China

## Abstract

**Background:**

Emerging evidences have shown that long noncoding RNA SPRY4-IT1 can be aberrantly expressed in human cancers, and it could be an unfavorable prognostic factor in cancer patients. However, the prognostic mechanism of SPRY4-IT1 is still unclear. This study is aimed at evaluating its potential predictive value for cancer prognosis.

**Methods:**

We thoroughly searched PubMed, Embase, Web of Science, and MEDLINE databases so as to explore the relationship between SPRY4-IT1 expression and cancer prognosis value. Then, TCGA datasets were used to validate the results of our meta-analysis.

**Results:**

In all, seventeen studies involving 1650 patients were included in this meta-analysis. Pooled results showed that high expression of SPRY4-IT1 was significantly correlated with poor OS (HR = 1.96, 95% confidence interval (CI) = 1.47‐2.62, *P* < 0.001) in cancer patients. Furthermore, exploration of TCGA dataset further validated that SPRY4-IT1 was aberrantly expressed in various cancers, which partially confirmed our results in this meta-analysis.

**Conclusions:**

The present systematic review and meta-analysis implicated that the aberrant expressions of lncRNA SPRY4-IT1 were strongly associated with clinical survival outcomes in various cancers and therefore might serve as a promising biomarker for predicting prognosis of human cancers.

## 1. Introduction

Nowadays, cancer is considered the leading cause of death worldwide and the occurrence of it is still increasing due to various risk factors [1]. In 2019, an estimated 1,762,450 new cancer patients were diagnosed in the US and 606,880 of them died of cancer, equivalent to nearly 1700 deaths a day [2]. Although the comprehensive treatments including surgery, chemotherapy, radiotherapy, and immunotherapy have made great progress, many patients in advanced clinical stage or with metastasis have lost the opportunity for surgery, become resistant to chemotherapy, and have poor prognosis. One of the important reasons for such a condition is the lack of effective early diagnosis indicators [3]. Therefore, it is very urgent and important to find the related biomarkers for cancer occurrence, development, and prognosis.

Long noncoding RNAs are RNA transcripts whose length is more than 200 nucleotides [4]. The functions of lncRNA may include transcriptional modulating, splicing regulating, posttranscriptional processing, chromatin remodeling, and regulation on protein-protein, protein-DNA, and protein-RNA interactions [5]. Recently, accumulating evidence has shown that certain lncRNAs are dysregulated in various cancers. Previous evidences [6, 7] suggest that lncRNA is significantly correlated with the growth, proliferation, invasion, metastasis, and angiogenesis of cancer cells. It can function as oncogene or tumor suppressor to regulate cancer-related signal pathway directly or indirectly and thereby affect the development of cancer. Therefore, lncRNA may have prognostic value in the regulation of carcinogenesis and cancer progression.

Recent studies have shown that long noncoding RNA SPRY4-IT1 has dysregulated expressions in a variety of tumors, including hepatocellular carcinoma [8], breast cancer [9], gastric cancer [10], cervical cancer [11], cholangiocarcinoma [12], pancreatic carcinoma [13], osteosarcoma [14], and colorectal cancer [15]. SPRY4-IT1 located on chromosome 5q31.3 is derived from an intron of the Sprouty 4 (SPRY4) gene, which was first reported in cutaneous melanoma and plays an important role in the growth, apoptosis, invasion, and migration of melanoma cells [16]. In addition, emerging studies have confirmed the importance of SPRY4-IT1 in the regulation of tumor-related signaling pathways, including AMPK [10], PI3K/Akt [17], Wnt/*β*-Catenin [18], and JAK2/STAT3. The expression level of SPRY4-IT1 in bladder cancer is significantly correlated with the advanced cancer stage, lymph node metastasis, and distant metastasis [19]. Furthermore, it is confirmed that the expression of epithelial-mesenchymal transition-related genes is modulated by alteration of SPRY4-IT1 expression [20]. In conclusion, SPRY4-IT1 may be a risk factor and a new potential therapeutic target for intervention against human malignant tumors. However, the prognostic value of SPRY4-IT1 is still indistinct because previous studies have been conducted with small sample sizes and controversial survival outcomes in the clinical settings. Therefore, we collected more eligible studies and conducted quantitative meta-analysis to further assess the prognostic significance of SPRY4-IT1 in various cancers.

## 2. Methods

### 2.1. Search Strategy

The present study was rigorously designed and reviewed according to the guidelines of the Preferred Reporting Items for Systematic Reviews and Meta-Analysis (PRISMA) statement checklist [21]. This systematic review and meta-analysis was registered (PROSPERO registration no. CRD42020167279) online. PubMed, Embase, Web of Science, and MEDLINE were comprehensively searched for eligible studies up to February 2020. We used the following: “long noncoding RNA SPRY4-IT1 OR SPRY4-IT1 RNA OR SPRIGHTLY lncRNA OR SPRY4-IT1 lncRNA OR SPRY4-IT1 OR SPRIGHTLY OR SPRY4 Intronic Transcript 1” AND “neoplasm OR neoplasia OR tumor OR cancer OR malignancy OR malignant OR carcinoma OR malignant neoplasm” AND “prognosis OR prognoses OR prognostic OR survival OR outcome OR OS” with language limited to English. Additionally, the citation lists of these retrieved articles were manually screened to ensure the sensitivity of the search strategy.

### 2.2. Inclusion and Exclusion Criteria

Inclusion criteria are as follows. (a) Cancer patients in the studies were confirmed through pathological examinations. (b) Patients were divided into high and low expression groups based on the cutoff values of SPRY4-IT1 expressions. (c) Sufficient data on the relationship between SPRY4-IT1 expression and overall survival (OS) were collected. (d) The effect size is evaluated by estimated hazard ratios (HRs) and the corresponding 95% confidence intervals (CIs).

Exclusion criteria are as follows: (a) studies without data related to the overall survival; (b) duplicate publications or repetitive analyses; (c) studies based on animal models or cell lines; and (d) reviews, letters to the editor, abstracts, and case report.

### 2.3. Data Extraction and Quality Assessment

All relevant articles were evaluated and extracted by two independent authors (HMW and KP). Any disagreements were consulted with the third investigator (HJL). For each study, the following items were extracted: first author, publication year, country, tumor type, sample type, sample size, number of high SPRY4-IT1 expression group and low SPRY4-IT1 expression group, tumor stage, cutoff value, detection method, metastasis, follow-up period, survival analysis type, and survival outcome. When more than one HR was reported, the most adjusted HR was extracted. All discrepancies between the two reviewers were resolved by consensus. The extracted HRs and 95% CI were standardized into the form of high SPRY4-IT1 expression versus low SPRY4-IT1 expression. If the results of OS were provided in the studies, multivariate analysis was first considered over univariate analysis. If Kaplan-Meier (K-M) curves were the only curves provided in certain studies, we would use the Get Data Graph Digitizer (Version 2.25) to calculate the pooled HRs and 95% CIs through indirect extraction from the plots [22]. If data of interest were not accessible, we would obtain the missing data from corresponding authors of enrolled articles. Since all studies included in our meta-analysis were cohort studies, the Newcastle–Ottawa Scale (NOS) was used to assess the quality of each by two independent authors (HMW and QZ). The NOS consists of three parts: selections, comparability, and measurement of outcomes. Studies with NOS scores being 6 or above were assigned as high methodological studies.

### 2.4. Validation by Reviewing Public Data

This study is in accordance with the publication guidelines from The Cancer Genome Atlas (TCGA). In total, we screened data of 14 types of cancers that have both SPRY4-IT1 expression data and survival data. The patients were divided into high and low SPRY4-IT1 expression groups according to the median value of SPRY4-IT1 expression. The survival analysis was calculated by the K-M method and logrank test [22].

### 2.5. Statistical Analysis

Stata SE12.0 and RevMan 5.3 software were used for statistical analysis. Pooled HRs and 95% CIs were obtained from the included studies. The HRs for OS and odds ratios (ORs) for clinicopathological parameter were statistically analyzed [23]. Chi-square-based *Q* test and *I*^2^ statistic were undertaken to assess the heterogeneity of the included trials. If *I*^2^ > 50% or *P* value < 0.05, the significant heterogeneity would be observed and the random-effects model would be applied. Otherwise, the fixed-effects model would be adopted. Publication bias was assessed by visual inspection of the Begg funnel plot. The stability of results was testified by a sensitivity analysis.

## 3. Results

### 3.1. Study Selection and Study Characteristics

**Figure 1:**
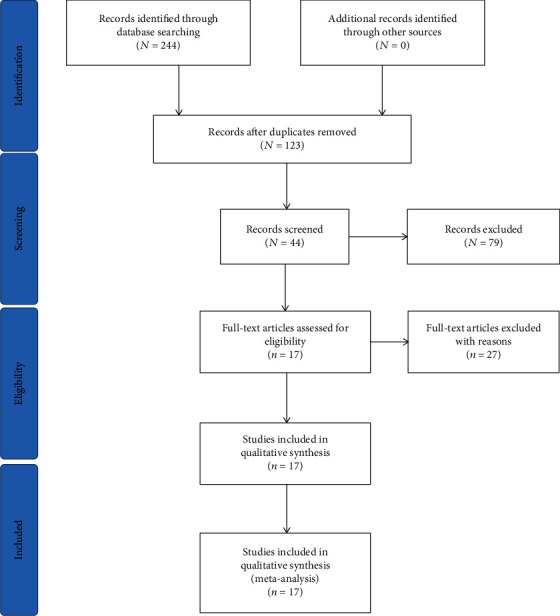
Flow diagram of the study selection process.

A total of 244 relevant articles in conformity with our search strategy were collected from the databases. After the duplications were removed, 123 publications that remained were screened of their titles and abstracts, and 79 publications were further excluded as they were reviews, letters to the editor, meeting abstracts, case reports, or irrelevant contents. The full texts of the remaining 44 publications were further examined, and 27 of them were excluded due to the lack of survival data. Ultimately, seventeen studies [12, 13, 15, 20, 24–36] involving 1650 patients were included in this meta-analysis. The flow diagram in Figure 1 illustrates the selection process.

These articles were published between 2014 and 2019 with the sample sizes ranging from 46 to 175. As shown in Table 1, all the studies were carried out in China and quantitative real-time reverse transcription PCR was used as the detection method. Sixteen studies used tissue samples for the detection of the expression of SPRY4-IT1, while one used blood samples. A total of 14 different types of tumors were included in these studies, including 2 cases of colon cancer, 1 case of cervical cancer, 2 cases of gastric cancer, 1 case of liver cancer, 1 case of ovarian cancer, 2 cases of lung cancer, 1 case of breast cancer, 1 case of esophageal cancer, 1 case of renal cancer, 1 case of bladder cancer, 1 case of cholangiocarcinoma, 1 case of melanoma cancer, 1 case of pancreatic carcinoma, and 1 case of glioma. Patients in all eligible studies were divided into high and low expression groups of SPRY4-IT1 according to the cutoff values. The follow-up time ranged from 60 to 80 months. Due to the low overall risk of correlation between SPRY4-IT1 expression and cancers in the included literatures, HRs in the cohort studies were similar to the RRs in the cohort studies mathematically.

### 3.2. Association between SPRY4-IT1 and OS

**Figure 2:**
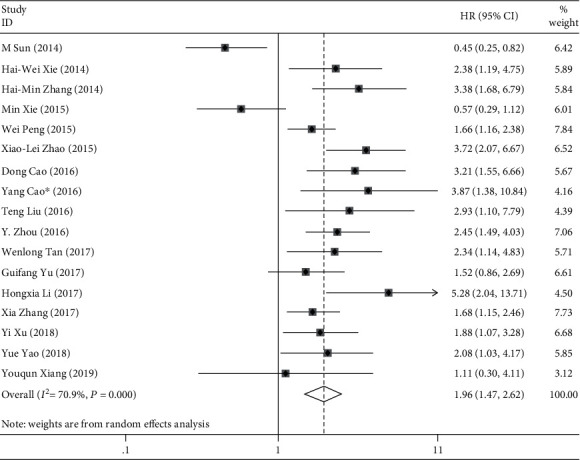
Forest plots of studies evaluating high SPRY4-IT1 expression in cancers for overall survival.

Seventeen studies investigated the relationship between SPRY4-IT1 expression and OS. There was a significant heterogeneity in these studies (*I*^2^ = 70.9%, *P* < 0.001); the random-effects model was used to calculate the HR and 95% CI. The results show that elevated SPRY4-IT1 expression predicts poor OS in cancers (HR = 1.96, 95% CI 1.47-2.62, *P* < 0.001) (Figure 2).

### 3.3. Subgroup Analysis of Association between SPRY4-IT1 and OS

According to the sample size (less than 90 or more), tumor type (digestive tumors or others), cutoff value (mean, median, or 2^-*ΔΔ*Ct^), and follow-up time (less than 60 or more), we conducted stratified analyses to confirm the relationship between SPRY4-IT1 expression and OS in different subgroups. As shown in Table 2, none of the subgroup analyses change the predictive value between SPRY4-IT1 and OS.

### 3.4. Correlation between SPRY4-IT1 and Clinicopathological Parameters

We summarized the correlations between SPRY4-IT1 and clinicopathological parameters including age, gender, LNM, and DM. The results of these analyses are presented in Table 3. According to the pooled ORs, the SPRY4-IT1 expression is irrelevant to age or gender. In addition, the pooled effects indicate that high SPRY4-IT1 expression is significantly correlated with LNM (OR = 1.982, 95% CI 1.112~3.534, *P* = 0.020), DM (OR = 1.736, 95% CI 1.193~2.525, *P* = 0.004), and tumor size (OR = 2.201, 95% CI 1.696~2.857, *P* < 0.001).

### 3.5. Sensitivity Analysis

**Figure 3:**
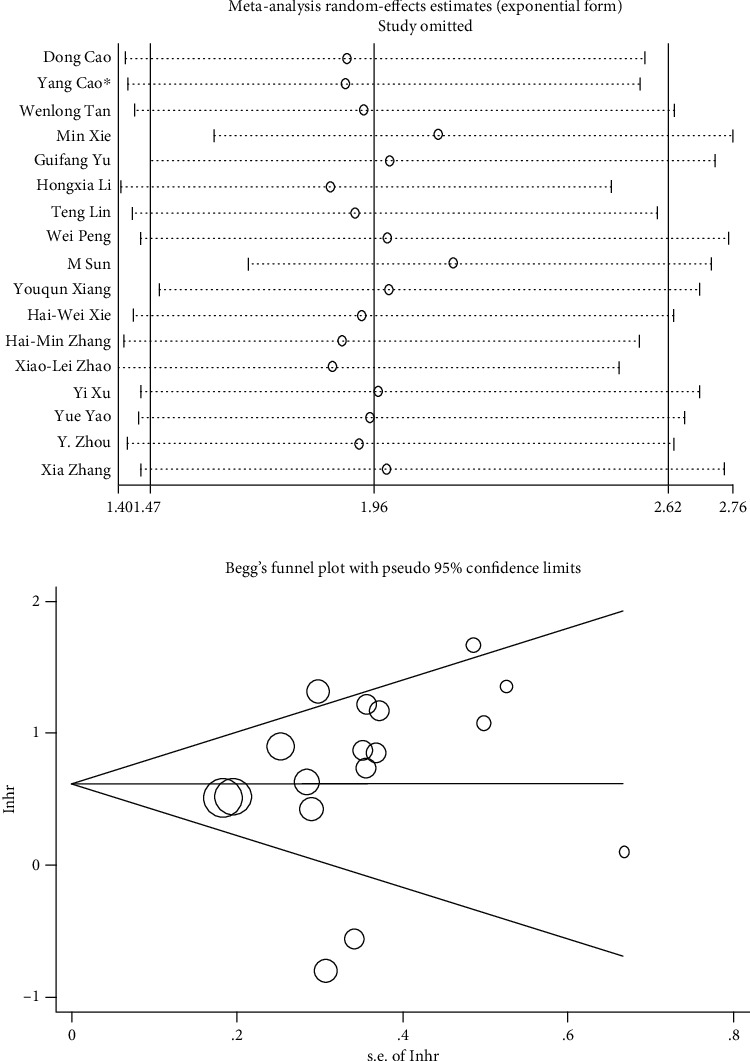
(a) Sensitivity analysis of pooled HR for overall survival. (b) Begg's funnel plot of SPRY4-IT1 for overall survival.

In order to assess the stability of the combined results of the association between SPRY4-IT1 expression and OS, sensitivity analysis was carried out by excluding studies one by one (Figure 3(a)). When “M Sun and Min Xie studies” were removed, the results showed that the high expression of SPRY4-IT1 still predicted the poor OS in cancers (HR = 2.17, 95% CI 1.86~2.54, *P* < 0.001) (Supplementary Figure 1).

### 3.6. Publication Bias

Begg's funnel plot was performed to evaluate publication bias (Figure 3(b)). The results show that there is no significant publication bias in the included studies (*P* = 0.232).

### 3.7. Validation of the Results in TCGA Dataset

**Figure 4:**
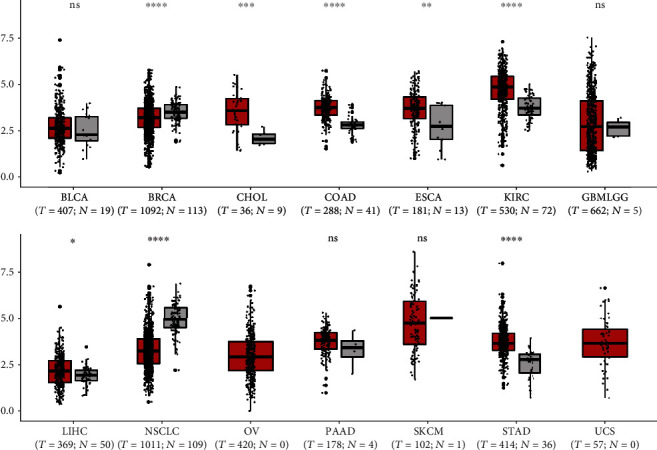
Validation of SPRY4-IT1 expression in various cancers in TCGA cohort. The expression levels of SPRY4-IT1 in BLCA (bladder urothelial carcinoma), BRCA (breast invasion carcinoma), CHOL (cholangiocarcinoma), COAD (colon adenocarcinoma), ESCA (esophageal carcinoma), KIRC (clear cell renal cell carcinoma), GBMLGG (glioma), LIHC (liver hepatocellular carcinoma), NSCLC (non-small-cell lung cancer), OV (ovarian cancer), PAAD (pancreatic ductal adenocarcinoma), SKCM (melanoma), and STAD (stomach adenocarcinoma).

**Figure 5:**
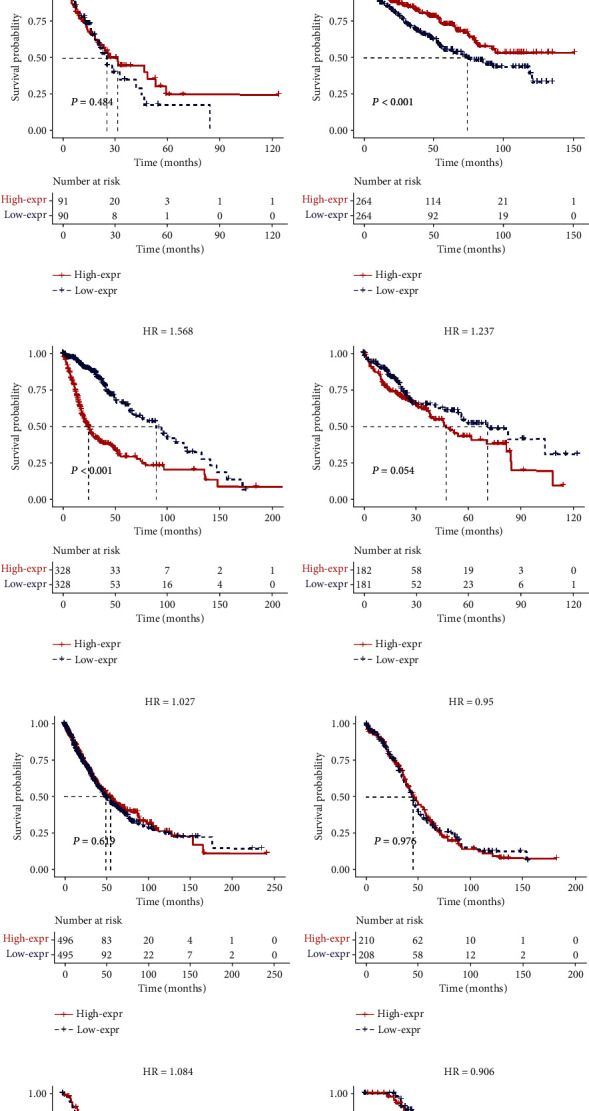
Survival plot of SPRY4-IT1 in TCGA cohort. Note: (a) BLCA, (b) BRCA, (c) CHOL, (d) COAD, (e) ESCA, (f) KIRC, (g) GBMLGG, (h) LIHC, (i) NSCLC, (j) OV, (k) PAAD, (l) SKCM, (m) STAD, and (n) UCS (*n* = 6219).

In order to further validate the result of our meta-analysis, TCGA datasets were analyzed to investigate whether SPRY4-IT1 could be involved in various cancers and influence patients' OS. In total, data of 6219 patients with 14 types of cancers were obtained. As shown in Figure 4, SPRY4-IT1 was aberrantly expressed in colorectal cancer, stomach adenocarcinoma, breast cancer, hepatocellular carcinoma, non-small-cell lung cancer, esophageal squamous cell carcinoma, clear cell renal cell carcinoma, and cholangiocarcinoma when compared with normal control (^∗^*P* < 0.05, ^∗∗^*P* < 0.01, ^∗∗∗^*P* < 0.001, ^∗∗∗∗^*P* < 0.0001, and ns means “not significant”). Moreover, the results also indicated that the upregulated SPRY4-IT1 expression may be closely relevant to poor overall survival in clear cell renal cell carcinoma and glioma (*P* < 0.001), which partially confirmed our results in this meta-analysis (Figure 5).

## 4. Discussion

Early studies suggested that lncRNA is a “cloning artifact” or “noise” after transcription [37]. Nowadays, mounting evidences showed that lncRNA acts as oncogene or tumor suppressor gene in a disease or tissue-specific manner in cancers [38]. Recent evidences revealed that many lncRNAs have abnormal expressions in various tumors, suggesting that lncRNAs play important roles in the pathogenesis and metastasis of tumors [39, 40]. They are also useful in tumor diagnosis and prognosis [41, 42]. For example, Sun et al. [7] established that LINC00301 is upregulated in both NSCLC tumorous tissues and cell lines and is correlated with worse prognosis in NSCLC patients. Therefore, it is necessary to understand the molecular mechanism of lncRNAs and identify sensitive, reliable biomarkers in order to develop a new targeted therapy for cancers.

SPRY4-IT1 has been demonstrated to be aberrantly expressed in multiple types of carcinoma cells. In our meta-analysis, SPRY4-IT1 expression levels were obviously upregulated in colorectal cancer, cervical cancer, hepatocellular carcinoma, ovarian cancer, melanoma, gastric cancer, breast cancer, esophageal squamous cell carcinoma, renal cell carcinoma, bladder cancer, cholangiocarcinoma, pancreatic ductal adenocarcinoma, glioma, and lung adenocarcinoma but downregulated in NSCLC. Data from TCGA database showed that the expression of SPRY4-IT1 was significantly decreased in NSCLC, which is coincident with the study by Sun et al. [32]. This disparity is also one of the interests and foci of our meta-analysis. Non-small-cell lung cancer (NSCLC) can be classified into adenocarcinoma, squamous cell carcinoma, adenosquamous carcinoma, large cell carcinoma, sarcomatoid carcinoma, pleomorphic carcinoma, etc. The most common subtype of NSCLC is lung adenocarcinoma, which accounts for approximately 40% of all lung cancers. Squamous cell carcinoma comprises 25–30% of all lung cancer cases. Large cell (undifferentiated) carcinoma accounts for 5–10% of lung cancers [33, 34]. The upregulation or downregulation of SPRY4-IT1 expression level in NSCLC may vary in patients of different pathological types. Moreover, the follow-up times and sample sizes of the two articles were different, and the patients in them came from different regions of China. Patients with non-small-cell lung cancer [32] were from Nanjing in southern China, while lung adenocarcinoma patients [26] were from Shandong in northern China. Demographic conditions also might influence the results of the experiments. In addition, different experimental conditions, experimental levels, experimenters, designs and implementation schemes, and experimental reagents may lead to different results. This systematic review and meta-analysis summarized and revealed that lncRNA SPRY4-IT1 is aberrantly expressed in various cancers, and this abnormality in NSCLC is also a new discovery worth noticing. Scientific research is a process of continuous exploration. Through systematic evaluation, we find that there are research differences, making it all the more necessary to continue the experimental exploration in this area. Moreover, we mainly explored the relationship between SPRY4-IT1 expression levels and cancer prognostic parameters. The pooled results revealed that high expression levels of SPRY4-IT1 predicted poorer OS (HR = 1.96, 95% CI 1.47-2.62, *P* < 0.001). Elevated SPRY4-IT1 expression was obviously correlated with advanced distant metastasis (OR = 1.736, 95% CI 1.193~2.525, *P* = 0.004), lymph node metastasis (OR = 1.982, 95% CI 1.112~3.534, *P* = 0.020), and tumor size (OR = 2.201, 95% CI 1.696~2.857, *P* < 0.001) [31]. Further studies on the mechanism indicated that the knockdown of SPRY4-IT1 could cause significant changes in cell proliferation, migration [23], colony formation [28], invasion, apoptosis, and epithelial-mesenchymal transition (EMT). In addition, subgroup analysis suggested that sample size, sample type, cutoff time, and follow-up time did not alter prognostic value of SPRY4-IT1 or the overall survival. Notably, the results from TCGA databases also indicated that the upregulated SPRY4-IT1 expression may be closely relevant to poor overall survival in clear cell renal cell carcinoma and glioma (*P* < 0.001), which partially identified our results in this meta-analysis.

Further experiments highlighted that SPRY4-IT1 may function as a ceRNA and sponge miRNA and thereby scaffolding target genes in cancers, such as miR-101-3p/EZH2 in cholangiocarcinoma [12], miR-101-3p/AMPK axis in gastric cancer [10], and miR-6882-3p/TCF7L2 in breast cancer cell stemness [9]. Yu et al. [28] reported that SPRY4-IT1 has interaction with EER*α*, and SPRY4-IT1 siRNA transfection HepG2 cells significantly inhibited mRNA and protein expression levels of ERR*α*in in HCC cell lines, thus influencing cell proliferation, colony formation, cell invasion, and migration [29]. Moreover, SPRY4-IT1 can interact with many signal pathways. Yao et al. [13] demonstrated that knockdown of SPRY4-IT1 expression could influence cell apoptosis partly via the Bcl-2/caspase-3 pathway in PANC1 and Capan-2 cells. Mechanism assays also noted that SPRY4-IT1 participated in the progression of thyroid cancer through targeting the TGF-*β*/Smad signaling pathway [43].

The highlights of our meta-analysis are as follows. Although previous studies have evaluated the clinicopathological significance and prognostic value of SPRY4-IT1 level in various cancers, this study is the first meta-analysis that combined data from a large published dataset with TCGA. We drew the box diagrams and the survival curves to present the results in a visual and intuitive way. Moreover, three literatures [3–5] reported the ratio in the form of low SPRY4-IT1 expression versus high SPRY4-IT1 expression; other studies reported the ratio reversely. In our study, the extracted HRs and 95% CI were standardized as high SPRY4-IT1 expression versus low SPRY4-IT1 expression. Therefore, the data is more rigorous and standardized, making a difference from previous reviews. In addition, we collected a larger size of samples, updated literatures, included more types of cancers, and used stringent inclusion criteria to ensure the reliability of the data. Considerable effort was made to conduct an extensive literature research.

Our research also has some limitations. First, this study was constrained to studies published in China only. Publication bias cannot be excluded. Second, the definition of SPRY4-IT1 cutoff values in each center is inconsistent. As the cutoff values in the selected literatures are classified according to medians, we also extract survival data in TCGA by selecting the median as the cutoff value. In the previous study, subgroup analysis proved that the method of dividing cutoff values was not a source of heterogeneity. This method may cause deviation. Hence, it is urgent to establish the standard and unified SPRY4-IT1 cutoff value. Further investigations involving the unified SPRY4-IT1 cutoff are needed to achieve more meaningful results. Third, some HRs are calculated by reconstructing the K-M curve, rather than directly from the original research, which would inevitably lead to possible deviations. Fourth, there was significant heterogeneity across the included studies in HR for OS (*I*^2^ = 70.9%, *P* ≤ 0.001). Despite the application of sensitivity analysis and subgroup analysis, the origin of heterogeneity could not be fully traced. Fifth, it is true that gastric cancer was included in this meta-analysis, but only data with gastric adenocarcinoma samples were found in TCGA database. The large majority (approximately 90%) of gastric cancers are adenocarcinomas, which arise from the glands of the most superficial layer, or the mucosa, of the stomach [44]. Therefore, if not specified otherwise, our discussion of gastric cancer (GC) mainly refers to adenocarcinomas [45]. However, there are still some limitations in using data for gastric adenocarcinoma in TCGA database to verify the results of gastric cancer in this meta-analysis. In the future, further clinical studies are still needed to verify these results.

In conclusion, the present systematic review and meta-analysis implicated that aberrant expression of lncRNA SPRY4-IT1 was strongly associated with clinical survival outcomes in various cancers and therefore might serve as a promising biomarker for predicting prognosis of human cancers. In the future, further clinical studies are still needed to demonstrate these results.

## Figures and Tables

**Table 1:** Summary of the main characteristics of the studies enrolled in the meta-analysis.

First author	Year	Country	Sample size	SPRY4-IT1 expression		Tumor type	Sample type	Follow-up time (month)	Tumor stage	Cutoff value	Detection method	Metastasis	Survival analysis	Outcome	NOS
				High	Low										
Dong Cao	2016	China	84	36	48	CRC	Tissue	36	TNM I-IV	2.87	qRT-PCR	DM	M	OS	7
Yang Cao	2016	China	100	46	54	Cervical cancer	Tissue	60	FIGO I-II	2.76	qRT-PCR	LNM	M	OS	8
Wenlong Tan	2017	China	106	58	48	CRC	Tissue	N/A	TNM I-IV	Mean	qRT-PCR	DM/LNM	M	OS	8
Min Xie	2015	China	61	30	31	Gastric cancer	Tissue	36	TNM I-IV	0.535	qRT-PCR	LNM	U	OS	6
Guifang Yu	2017	China	82	45	37	HCC	Tissue	60	TNM I-IV	Median	qRT-PCR	N/A	U	OS	8
Hongxia Li	2017	China	124	62	62	Ovarian cancer	Tissue	60	FIGO I-IV	Median	qRT-PCR	LNM	M	OS	8
Teng Liu	2016	China	70	32	38	Melanoma	Blood	60	TNM I-IV	2.64	qRT-PCR	N/A	M	OS	8
Wei Peng	2015	China	175	98	77	Gastric cancer	Tissue	60	TNM I-IV	0.778	qRT-PCR	DM	M	OS	8
M Sun	2014	China	121	60	61	NSCLC	Tissue	36	TNM I-III	Median	qRT-PCR	LNM	M	OS	9
Youqun Xiang	2019	China	102	52	50	Breast cancer	Tissue	N/A	TNM I-III	Mean	qRT-PCR	LNM	M	OS	8
Hai-Wei Xie	2014	China	92	46	46	ESCC	Tissue	60	TNM I-IV	4.52	qRT-PCR	LNM	M	OS	8
Hai-Min Zhang	2014	China	98	52	46	CCRCC	Tissue	60	TNM I-IV	3.38	qRT-PCR	DM/LNM	M	OS	8
Xiao-Lei Zhao	2015	China	68	38	30	Bladder cancer	Tissue	60	TNM I-IV	3.68	qRT-PCR	LNM	M	OS	7
Yi Xu	2018	China	70	41	29	CCA	Tissue	60	TNM I-IV	2.65	qRT-PCR	NA	M	OS	7
Yue Yao	2018	China	46	26	20	PDAC	Tissue	61	TNM I-IV	3.57	qRT-PCR	NA	M	OS	7
Y. Zhou	2016	China	163	81	82	Glioma	Tissue	N/A	TNM I-IV	Median	qRT-PCR	NA	M	OS	7
Xia Zhang	2017	China	88	44	44	LUAD	Tissue	60	N/A	Median	qRT-PCR	NA	M	OS	7

Abbreviations: CRC: colorectal cancer; HCC: hepatocellular carcinoma; NSCLC: non-small-cell lung cancer; ESCC: esophageal squamous cell carcinoma; CCRCC: clear cell renal cell carcinoma; CCA: cholangiocarcinoma; PDAC: pancreatic ductal adenocarcinoma; LUAD: lung adenocarcinoma; DM: distant metastasis; TNM: tumor node metastasis; FIGO: International Federation of Gynecology and Obstetrics; LNM: lymph node metastasis; N/A: not available; qRT-PCR: reverse transcription quantitative polymerase chain reaction; M: multivariate; U: univariate; NOS: Newcastle–Ottawa Scale; OS: overall survival.

**Table 2:** Stratified analyses of the pooled HRs of overall survival by sample size, tumor type, cutoff value, and follow-up time.

		Random-effects model		Fixed-effects model		Heterogeneity	
Analysis	No. of studies	HR (95% CI)	*P*	HR (95% CI)	*P*	*I* ^2^	*P*
Subgroup 1: Sample size < 90	8	1.896 (1.313~2.763)	<0.001	1.846 (1.501~2.272)	<0.001	66.90%	0.004
Sample size ≥ 90	9	2.046 (1.287~3.252)	0.002	1.863 (1.513~2.293)	<0.001	76.40%	<0.001
Subgroup 2: Digestive system	9	1.913 (1.381~2.650)	<0.001	1.869 (1.542~2.266)	<0.001	62.40%	0.006
Others	8	2.066 (1.196~3.567)	0.009	1.834 (1.462~2.302)	<0.001	79.20%	<0.001
Subgroup 3: Cutoff value: mean	5	2.543 (1.795~3.604)	<0.001	2.560 (1.884~3.477)	<0.001	19.50%	0.291
Cutoff value: median	7	1.491 (0.867~2.562)	0.149	1.479 (1.196~1.828)	<0.001	83.30%	<0.001
Cutoff value: 2^-*ΔΔ*Ct^	5	2.194 (1.595~3.017)	<0.001	2.084 (1.589~2.734)	<0.001	15.30%	0.317
Subgroup 4: Follow-up time < 60	3	0.924 (0.288~2.966)	0.894	0.831 (0.567~1.217)	0.341	89.10%	<0.001
Follow-up time ≥ 60	11	2.248 (1.789~2.826)	<0.001	2.108 (1.771~2.509)	<0.001	34.50%	0.122

**Table 3:** Correlation between lncRNA SPRY4-IT1 expression and clinicopathological parameters for cancers.

Clinicopathological parameters	No. of studies	Pooled OR (95% CI)	*P*	Model	Heterogeneity		
					Chi^2^	*P* value	*I* ^2^
Age	6	0.889 (0.626~1.262)	0.512	Fixed	1.38	0.926	0.00%
Gender	14	0.994 (0.797~1.239)	0.954	Fixed	8.41	0.816	0.00%
LNM	13	1.982 (1.112~3.534)	0.020	Random	60.55	<0.001	80.20%
DM	5	1.736 (1.193~2.525)	0.004	Random	30.99	<0.001	87.10%
Tumor size	9	2.201 (1.696~2.857)	<0.001	Fixed	9.96	0.268	19.7%

## Data Availability

N/A.

## Supplementary Materials

Supplementary Materials Supplementary Figure 1: forest plots of studies evaluating high SPRY4-IT1 expression in cancers for overall survival. (“M Sun and Min Xie studies” were removed).
