# Supplementary material for: Prognostic Value of Long Noncoding RNA SPRY4-IT1 on Survival Outcomes in Human Carcinomas: A Systematic Review and Meta-Analysis with TCGA Database
Source: Biomed Res Int. 2020 Nov 1;2020:5868602. doi: 10.1155/2020/5868602 (PMC7652610; doi:10.1155/2020/5868602)

Supplementary Fig. 1 Forest plots of studies evaluating high SPRY4-IT1 expression in cancers for overall survival.(M Sun and Min Xie studies" were removed)

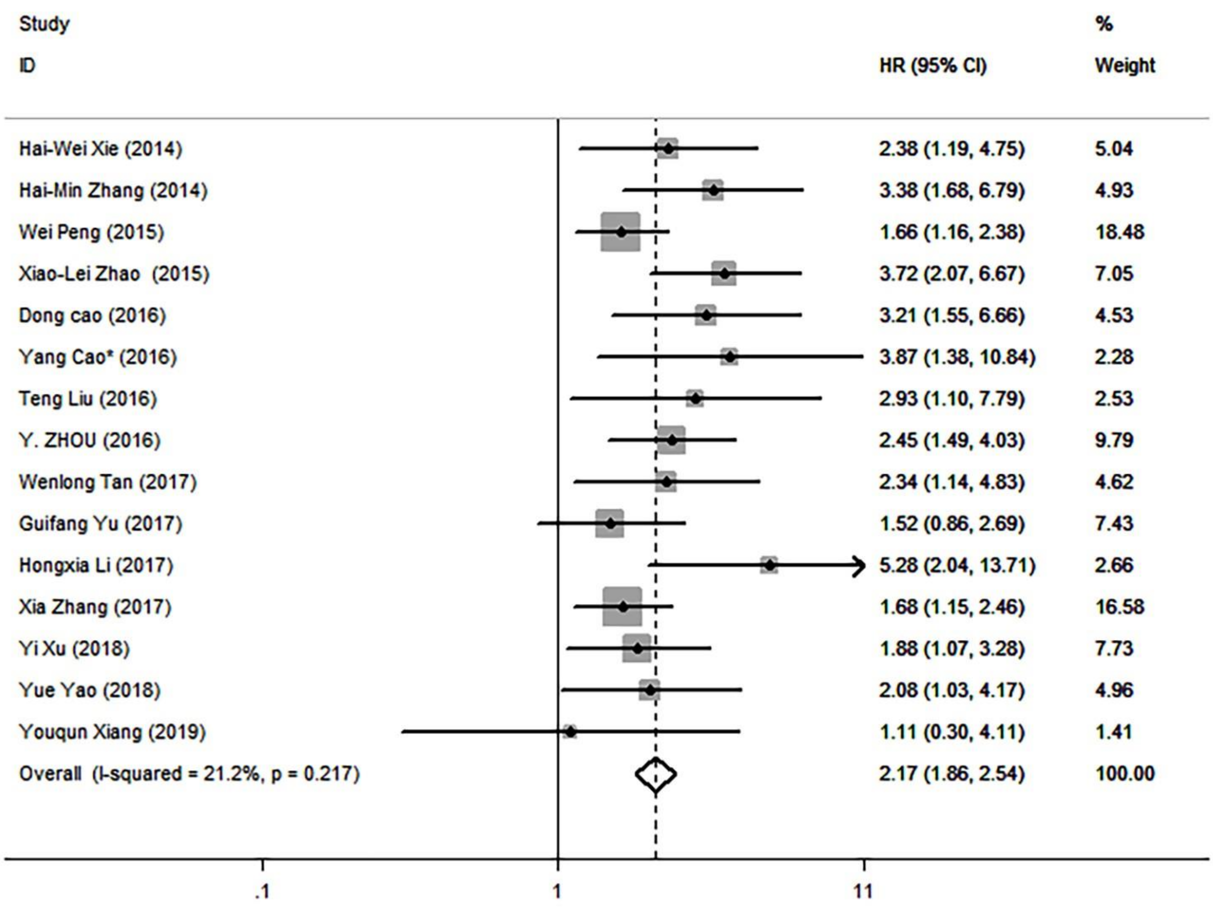

Supplement: Supplementary Materials — Supplementary Figure 1: forest plots of studies evaluating high SPRY4-IT1 expression in cancers for overall survival. (“M Sun and Min Xie studies” were removed). [file 5868602.f1.pdf]
